# Supplementary material for: Yiai Fuzheng decoction inhibits triple-negative breast cancer by remodeling the immune microenvironment
Source: Front Immunol. 2025 Sep 30;16:1615631. doi: 10.3389/fimmu.2025.1615631 (PMC12518410; doi:10.3389/fimmu.2025.1615631)
Supplement: Supplementary file 2 [file DataSheet1.zip › Supplementary File 2/Treatment/D_vs_H/02_PCA/All_Groups/All_Combinaiton/PC12_Score_2D.pdf]

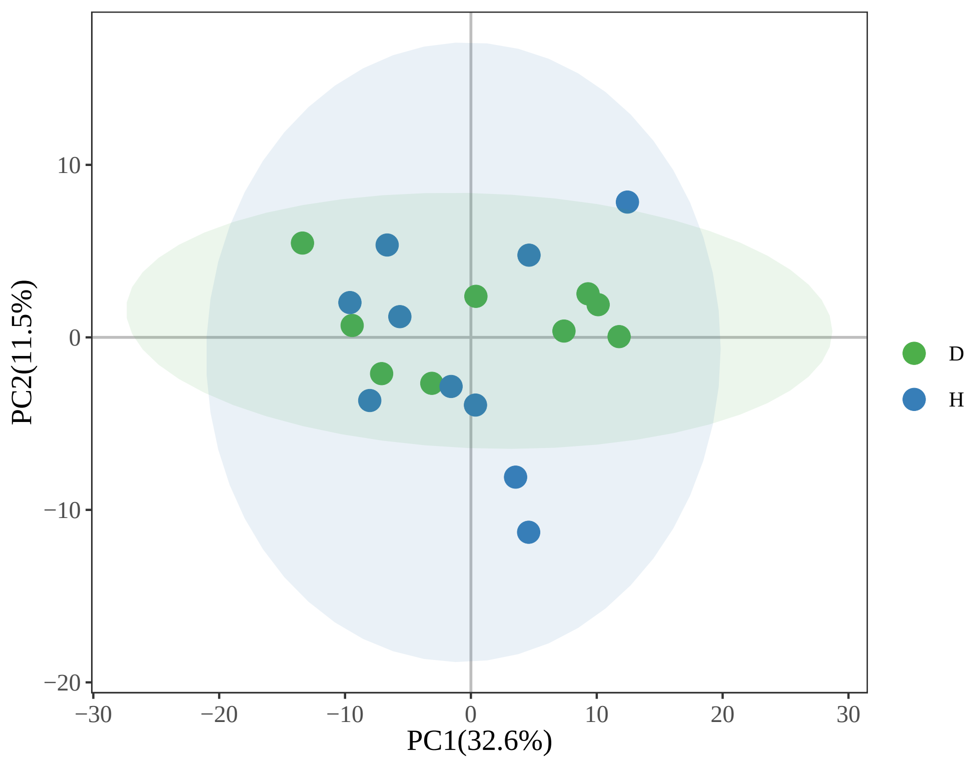

10
0
−10
−20
−30
−20
−10
0
10
20
30
PC1(32.6%)
PC2(11.5%)
D
H
